# PINA 3.0: mining cancer interactome

**DOI:** 10.1093/nar/gkaa1075

**Published:** 2020-11-24

**Authors:** Yang Du, Meng Cai, Xiaofang Xing, Jiafu Ji, Ence Yang, Jianmin Wu

**Affiliations:** Center for Cancer Bioinformatics, Key Laboratory of Carcinogenesis and Translational Research (Ministry of Education), Peking University Cancer Hospital & Institute, Beijing 100142, China; Institute of Systems Biomedicine, Department of Medical Bioinformatics, School of Basic Medical Sciences, Peking University Health Science Center, Beijing 100191, China; Department of Gastrointestinal Translational Research, Key Laboratory of Carcinogenesis and Translational Research (Ministry of Education), Peking University Cancer Hospital & Institute, Beijing 100142, China; Gastrointestinal Cancer Center, Key Laboratory of Carcinogenesis and Translational Research (Ministry of Education), Peking University Cancer Hospital & Institute, Beijing 100142, China; Institute of Systems Biomedicine, Department of Medical Bioinformatics, School of Basic Medical Sciences, Peking University Health Science Center, Beijing 100191, China; Center for Cancer Bioinformatics, Key Laboratory of Carcinogenesis and Translational Research (Ministry of Education), Peking University Cancer Hospital & Institute, Beijing 100142, China; Peking University International Cancer Institute, Peking University, Beijing 100191, China

## Abstract

Protein–protein interactions (PPIs) are crucial to mediate biological functions, and understanding PPIs in cancer type-specific context could help decipher the underlying molecular mechanisms of tumorigenesis and identify potential therapeutic options. Therefore, we update the Protein Interaction Network Analysis (PINA) platform to version 3.0, to integrate the unified human interactome with RNA-seq transcriptomes and mass spectrometry-based proteomes across tens of cancer types. A number of new analytical utilities were developed to help characterize the cancer context for a PPI network, which includes inferring proteins with expression specificity and identifying candidate prognosis biomarkers, putative cancer drivers, and therapeutic targets for a specific cancer type; as well as identifying pairs of co-expressing interacting proteins across cancer types. Furthermore, a brand-new web interface has been designed to integrate these new utilities within an interactive network visualization environment, which allows users to quickly and comprehensively investigate the roles of human interacting proteins in a cancer type-specific context. PINA is freely available at https://omics.bjcancer.org/pina/.

## Graphical Abstract

**Graphical Abstract:**
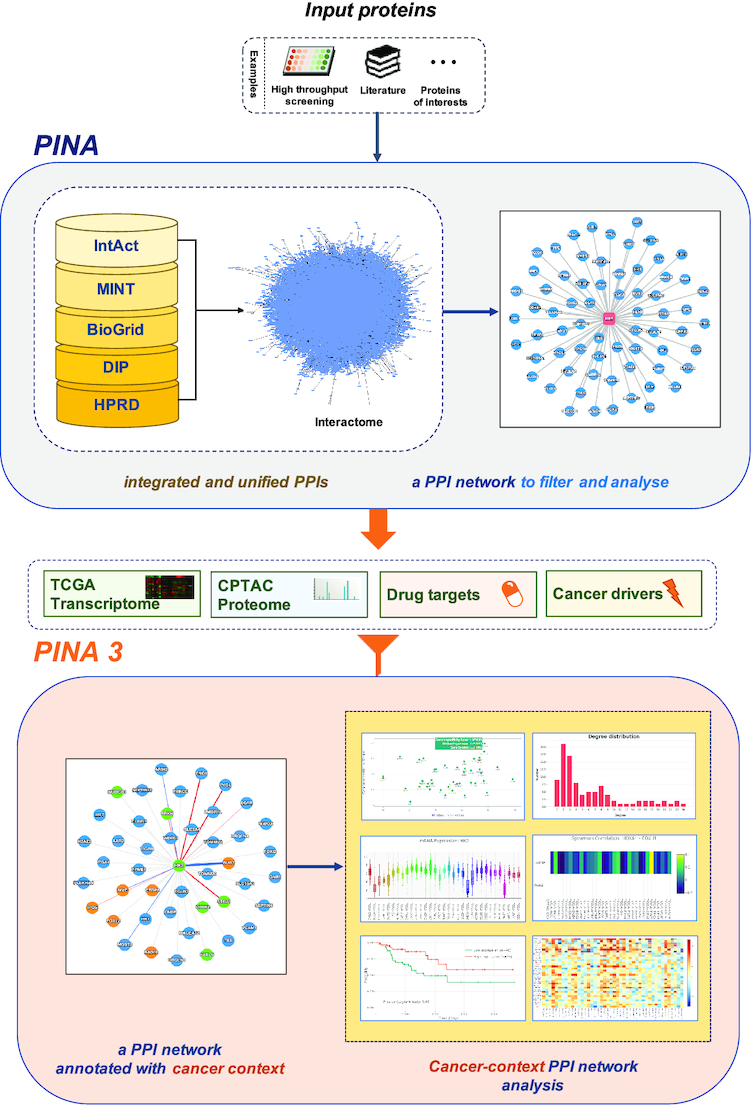
A schematic overview of PINA 3.0.

## INTRODUCTION

Protein–protein interactions (PPIs) are crucial for exerting biological functions in most cellular processes. With the quick accumulation of experimental PPI data, human protein interactome has been utilized in a variety of disease research (1–3). However, human proteins could have different interacting partners in different contexts (tissues, cell types, disease conditions) (4,5), which is important for understanding their functions within a specific context and identifying associations with the context-related phenotypes (6). Although this information is extremely valuable, a context-specific view of PPI networks has been rarely available.

Building a context-specific PPI network by experimental approaches is certainly precious. For example, Li *et al.* (7) detected a set of 397 high-confidence lung cancer-associated PPIs between 83 genes with a high frequency of genomic alterations in lung cancer, and discovered PPI network-implicated tumor vulnerabilities for therapeutic interrogation in lung cancer. However, it remains infeasible to systematically identify endogenous PPIs at protein interactome level for hundreds of pathological and molecular tumor types/subtypes. Thus, it is more common to predict context-specific PPIs based on expression profiles. Efforts have been made to generate context-based PPI networks including HIPPIE (8), IID (9), TissueNet (10), MyProteinNet (11) and HURI (12). However, context-specific PPIs in these databases were mostly inferred based on gene expression profiles of normal human tissues from the GTEx program (13) or the HPA project (14), comprehensive resources to investigate cancer contexts for human PPIs remain limited (15).

Recently, a massive amount of cancer sequencing and molecular profiling datasets have been publicly available from several international consortiums (16–18), which enables inferring tumor type-specific contexts for human reference interactomes, and offers unparalleled opportunities to reveal PPIs with functional significance and therapeutic implications, e.g. targeting interacting partners of hard-to-drug tumor suppressors. The Cancer Genome Atlas (TCGA) released RNA-seq data for ∼10 000 tumor samples across 33 cancer types, and the Clinical Proteomic Tumor Analysis Consortium (CPTAC) is rapidly accumulating proteome and phosphoproteome data for TCGA sequenced tumor samples as well as new samples, across tens of cancer types. Moreover, associating expression profiles with the accompanied patient outcome from these large cohort studies will help to indicate the functions of PPIs in tumorigenesis and progression. Taken together, characterizing tumor type-specific context for a given PPI network could be with great functional and therapeutic implications.

Thus, we release PINA version 3.0 to facilitate PPI network analysis within the cancer context. Using the integrated TCGA and CPTAC datasets, we developed several new utilities to infer proteins with tumor type-specific expression, to investigate expression correlations of interacting proteins across tumor types, to indicate interacting proteins associated with patient survival, to highlight mutational cancer drivers and therapeutic targets in a given PPI network. Furthermore, a brand-new web interface has been provided to integrate these new functions within an interactive network visualization environment, which allows users to quickly and comprehensively investigate the roles of human interacting proteins across tumor types in PINA version 3.0.

## MATERIALS AND METHODS

### PPI data update

PPI datasets were downloaded from five manually curated databases including IntAct (version 4.2.15) (19), MINT (download on 21 May 2020) (20), BioGRID (version 3.5.185) (21), DIP (version 20170205) (22) and HPRD (release 9) (23), and unified as described in the previous versions (24,25) to build a non-redundant PPI database for seven model organisms: *Homo sapiens*, *Mus musculus*, *Rattus norvegicus*, *Drosophila melanogaster*, *Caenorhabditis elegans, Saccharomyces cerevisiae* and *Arabidopsis thaliana*. All PPI data were stored in a MySQL relational database, and statistics of the current PINA release is available at https://omics.bjcancer.org/pina/stats.action.

### Integrated cancer datasets

Cancer transcriptomic profiles (26) were downloaded from the Genomic Data Commons (GDC) portal of TCGA (version 20190101). The batch-corrected and upper quartile normalized RSEM measurements were log2 transformed for further processing. Cancer proteomic profiles were downloaded from CPTAC data portal (version 20200511). The relative abundance of proteins generated by the Common Data Analysis Pipeline (CDAP) (27) was subjected to quantile normalization using *normalizeQuantiles* function implemented in R package limma (28) v3.36.1. Both mRNA and protein expression profiles were filtered by removing genes with zero or *NA* values in >80% samples in a dataset. Clinical information (survival time, tumor site, age, ethnicity and grade) were downloaded from both GDC and CPTAC for corresponding samples with molecular data. Only primary tumors were included in the PINA database.

### Tumor type-specific prognosis biomarkers

We used R package ‘survival’ (version 2.43.3) for Kaplan–Meier survival analysis. Samples were stratified into two groups: high expression versus low expression using six pairs of cutoffs for users’ choice (90% quantile versus 10% quantile, 80% quantile versus 20% quantile, 70% quantile versus 30% quantile, 75% quantile versus 25% quantile, 60% quantile versus 40% quantile and 50% quantile versus 50% quantile). Overall survival (OS) was used as the clinical endpoint. *P*-value was calculated by log-rank test for Kaplan–Meier analysis, and a gene with *P*-value less than 0.05 was considered as a candidate prognosis marker in a given tumor type.

### Identification of genes with tumor type-specific expression specificity

Tumor type specificity score was calculated using the method reported by Sonawane *et al.* (29), which compared the median expression level of a gene in a given tumor type to the median and interquartile range (IQR) of its expression across all tumor types. Specificity scores of each gene were calculated for mRNA and protein expression datasets respectively.

### Annotations of cancer driver genes and therapeutic targets

Cancer driver genes were obtained from a recent TCGA pan-cancer analysis (30), which characterized 9423 tumor exomes (comprising all 33 TCGA projects) and identified 299 mutational driver genes with implications regarding their anatomical sites and cancer types, including 258 genes from a systematic approach and 41 genes recovered after manual curation of previous TCGA marker papers of each individual study. Drugs and their potential targets were downloaded from the Genomics of Drug Sensitivity in Cancer (GDSC) release 8.3, which have been screened in >1000 human cancer cell lines in a previous pharmacogenomics study (31); and 812 cell lines were mapped to 30 cancer types based on TCGA classification. The pan-cancer information and links will be shown for a normal PPI network, while tumor type-specific information will be provided for a cancer-context PPI network.

### Website implementation

Since the last update of PINA, significant advances have been achieved in web technologies and network visualization (32). To provide better visualization effects in modern Internet browsers, we implemented a new web interface using HTML5 and open-source templates based on Bootstrap4 (http://getbootstrap.com/). The web application was built using Java Server Pages (JSP) technology hosted by an Apache Tomcat web server. Open-access web frameworks including Spring (https://spring.io/) and MyBatis (https://github.com/mybatis/mybatis-3/) were utilized to improve the sustainability and reliability of the web services.

PPI network visualization was mainly implemented using Cytoscape.js (32) (http://js.cytoscape.org/), and customized network functions were developed using native JavaScript libraries and JQuery (http://jquery.com). We implemented interactive plots using Plotly.js (https://github.com/plotly/plotly.js/), and dynamic tables using DataTables.js (https://www.datatables.net/).

## RESULTS

**Figure 1.**
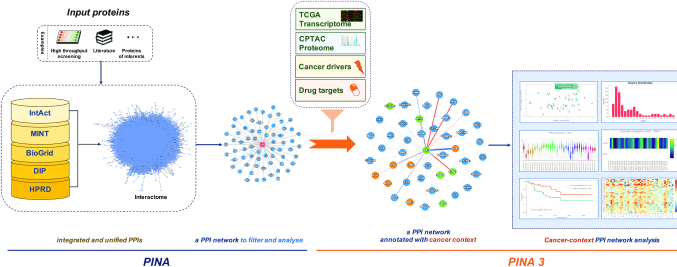
A schematic overview of PINA 3.0. PPIs were integrated and unified from five public databases to generate protein interactomes, which will be used to construct a user PPI network for input proteins of interests. PINA 3.0 enables cancer context-specific network annotation, visualization and analysis for a user PPI network, based on the integrated cancer datasets.

PINA 3.0 integrated non-redundant human PPIs with RNA-seq profiles of 9870 tumors across 33 cancer types from TCGA, and mass spectrometry-based proteomic profiles of 936 tumors across eight cancer types from CPTAC, to enable tumor type context-specific PPI network analysis. The schematic overview of PINA 3.0 is illustrated in Figure 1. The detailed documentation and a step-by-step case study tutorial are provided on the PINA website.

### New web interface with enhanced network visualization and interactive data reporting

**Figure 2.**
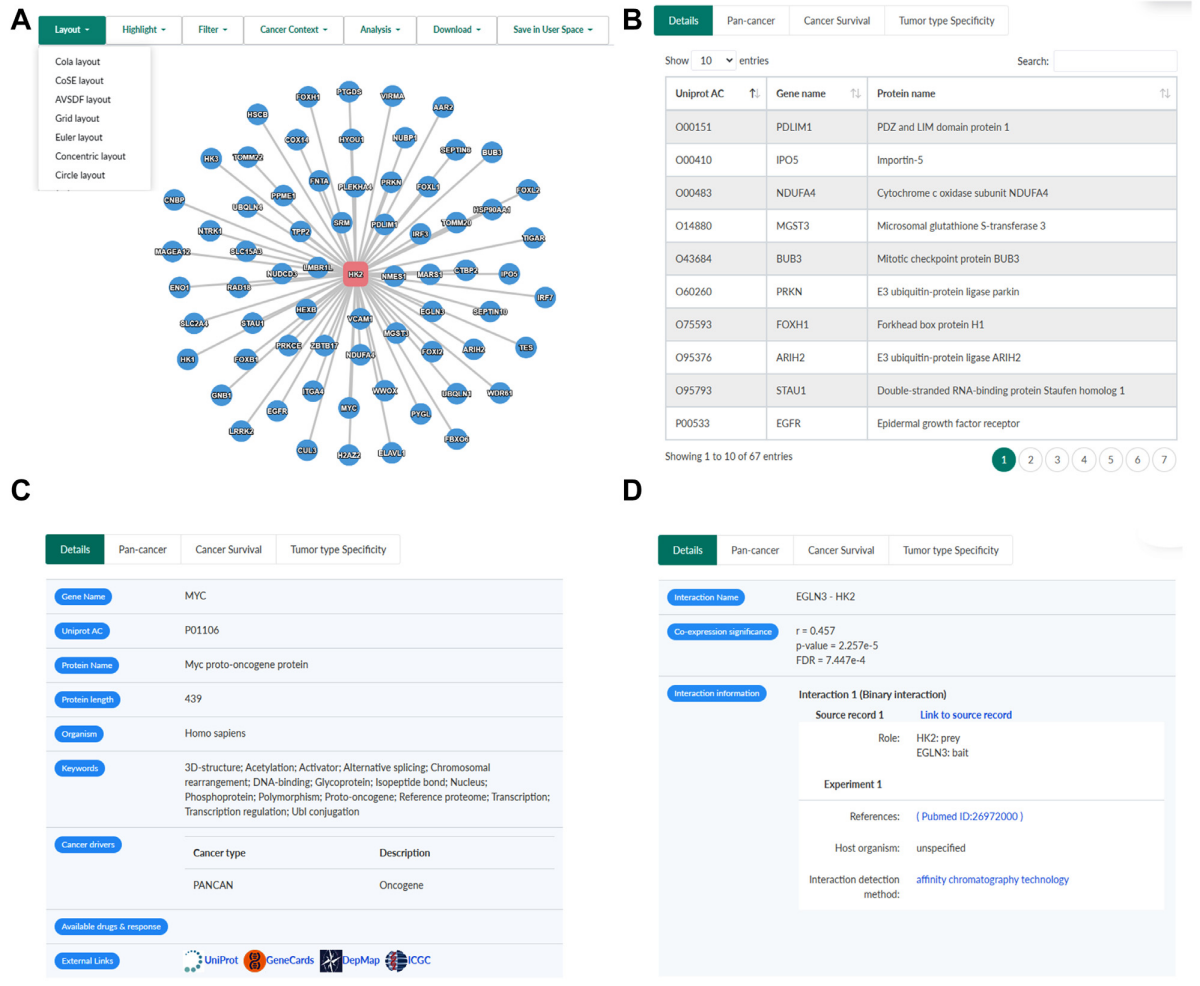
Enhanced network visualization and data reporting. A newly designed web interface consists of two panels: a graphical network-view panel (**A**) and a network-details panel showing respective details for an analyzed PPI network (**B**), a selected node (**C**) and a selected edge (**D**) correspondingly. Co-expression significance for a selected edge will only be available after cancer context annotated. Contents of ‘Tumor type specify’, ‘Pan-Cancer’ and ‘Cancer Survival’ tabs are shown in Figure 3C, D, F correspondingly.

We re-designed and implemented a brand new web interface using modern JavaScript libraries in this release, to improve network visualization and data reporting. After querying a network, all related information will be shown within a single web page consisting of two panels: a graphical network-view panel (Figure 2A) and a network-details panel (Figure 2B–D).

The network-view panel depicts proteins and their interactions as nodes and edges respectively with a force-direct layout by default. A toolbar menu is provided for changing network layout, highlighting proteins of interests, filtering interactions, building a cancer-context PPI network, presenting links to analysis tools, downloading network in multiple file formats, and saving network in the user space for long-term access. The newly developed *highlight* function enables users to highlight single protein, common interacting proteins, and cancer-related proteins (cancer drivers and therapeutic targets), which will be useful to efficiently identify proteins of interests in a big PPI network.

The network-details panel presents diverse rich information of interactors and interactions in a network. It consists of four tabs including network details (Figure 2B) using a sortable and searchable table, and a number of newly introduced cancer utilities (described in the next section). This panel is highly interactive with the network-view panel, by dynamic showing corresponding information upon clicking a node (Figure 2C) or an edge (Figure 2D) in the network-view panel.

### Novel features to help reveal tumor type-specific insights from PPI networks

**Figure 3.**
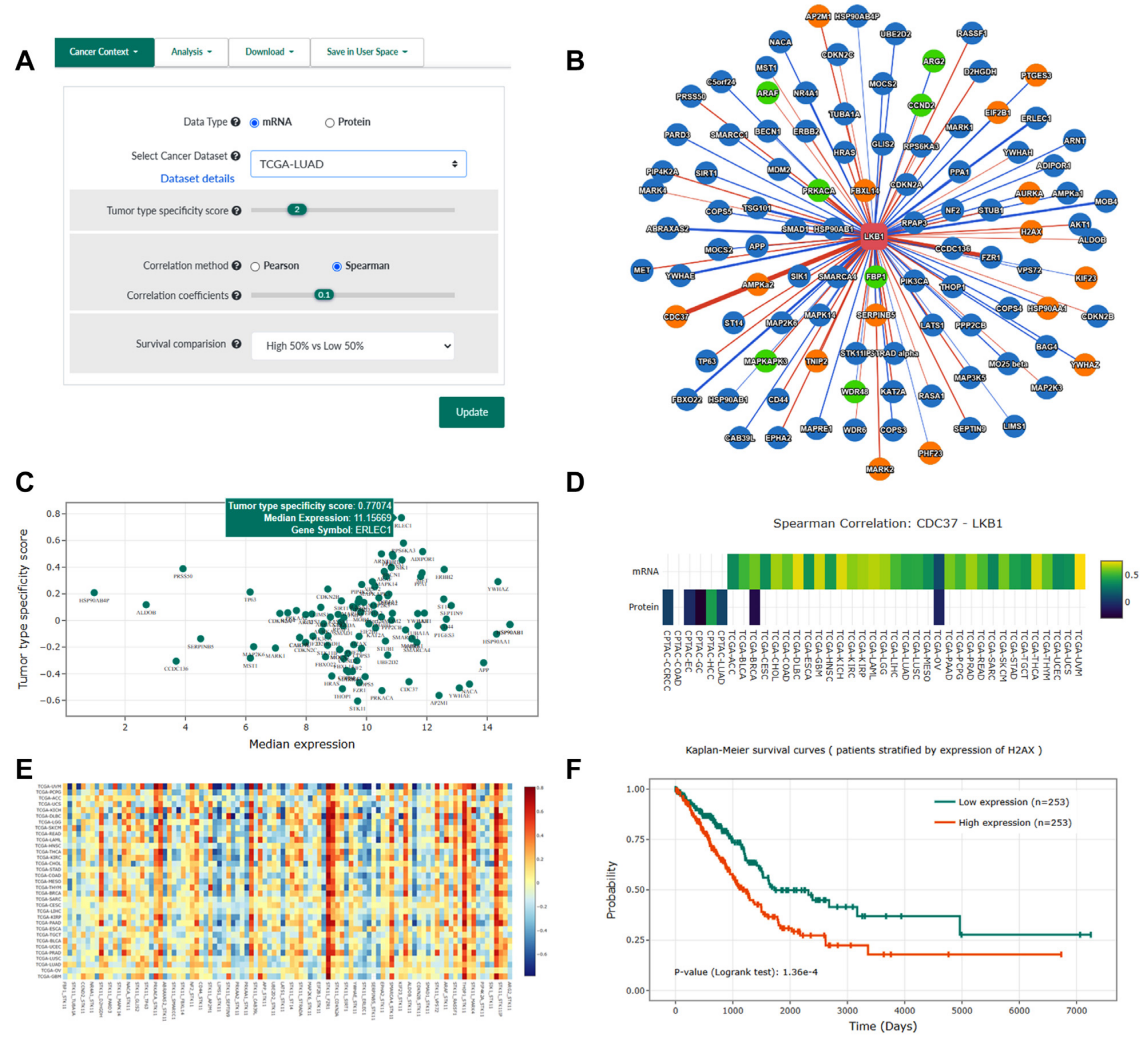
New features for cancer context annotation and analysis. (**A**) Query parameters to specify a cancer type context to annotate for a PPI network. (**B**) An example cancer-context PPI network of LKB1 using LUAD (Lung adenocarcinoma) mRNA profiles with parameters shown in (A). Candidate prognosis biomarkers are indicated as orange (poor prognosis) and green (good prognosis) nodes. Positive correlations with statistical significance between the expression levels of interacting proteins are represented as blue edges, while negative correlations are indicated as red edges. Edge width is proportional to the correlation coefficient. (**C**) A scatter plot illustrating relationships between the median expression of each gene in this network and their tumor type-specificity scores. (**D**) Correlation coefficients of mRNA expression levels and relatively protein abundance for the selected edge ‘LKB1-CDC37’ across all integrated tumor types. (**E**) Correlation coefficients of mRNA expression levels between all interaction pairs in the example network across cancer types. (**F**) A Kaplan–Meier survival curve showing significant survival differences between patients with high and low expression of *H2AX*.

We integrated TCGA and CPTAC datasets and developed new utilities to provide a ‘cancer context’ for a PPI network, which can be dynamically inferred through the network menu (‘Cancer Context’ tab, Figure 3A). A cancer-context PPI network provides multiple novel features to help reveal tumor type-specific insights from the original PPI network, as shown in the following sub-sections.

#### Cancer driver genes and therapeutic targets

Associating undruggable tumor suppressors with therapeutic targets in their interacting partners could inform cancer treatment options, thus both pan-cancer and cancer type-specific annotations of cancer driver genes and potential therapeutic targets were integrated into PINA 3.0. By clicking a node in a normal PPI network, pan-cancer information regarding cancer driver genes and available drugs (links to GDSC pan-cancer pharmacogenomic analysis results) will be shown in the network-details panel (‘Details’ tab, Figure 2C). For a cancer-context PPI network, the information and links will be changed according to the specified tumor type. Besides, PINA 3.0 provides the *highlight* function in the network menu (Figure 2A) to quickly identify all cancer driver genes and candidate therapeutic targets in a given PPI network.

#### Tumor type-expression specificity

Tissue-specific functions are largely mediated by interactions between tissue-specific proteins and uniformly expressed cellular members (12), and molecular profiles of tumors typically exhibit a cell-of-origin pattern (26), thus novel insights could be obtained from interacting proteins showing tumor type-expression specificity. This analysis also complements recent systematic efforts (33,34) in investigating how tissue-specific genes interact with other genes to mediate cancer-specific functions. Tumor type specificity scores were pre-calculated for each gene to represent the level of deviation of expression in a given tumor type compared to the full spectrum of tumor types. The cutoff of specificity score was set as 2 for mRNA expression levels (Supplementary Figure S1A–C), as suggested in previous studies(12,29). Genes having a specificity score >2 were considered as highly-expressed in the analyzed tumor type, and genes having a score ≤ 2 were considered as lowly-expressed (Figure 3B). As the proteomic datasets that PINA integrated were profiled by iTRAQ or TMT labelling methods, protein abundances were quantified relative to pooled samples or paired normal tissues. This resulted in a distribution different from mRNA levels, thus we set the cutoff as 0.5 by default for protein abundances, to have reasonable numbers of proteins with tumor type-expression specificity in each dataset (Supplementary Figure S1D–F). Users can also modify this cutoff at their will during analysis of a PPI network (Figure 3A). Moreover, a scatter plot will be shown in the network details panel (‘Tumor type Specificity’ tab) to illustrate the relationships between the median expression and the tumor type-specificity scores for a PPI network (Figure 3C).

#### Expression correlations of interacting proteins across tumor types

Gene co-expression analysis is valuable to identify functionally associated genes, and disease candidate genes. Thus, we pre-calculated the Pearson and Spearman correlation coefficients between all pairs of interacting proteins in PINA, for all integrated tumor types respectively. By clicking an edge in a network, a heatmap will be shown in the network-details panel (‘Pan-cancer’ tab) to present correlation coefficients between expression levels (mRNA and protein abundance) of interacting proteins across all integrated cancer datasets (Figure 3D). Two-tailed t-test will be applied to evaluate the statistical significance of correlation in a specific tumor type, with *P-*values corrected by the Benjamini-Hochberg method, which takes all PPIs in the analyzed network into account. Positive correlations and negative correlations with statistical significance (FDR < 0.05) between pairs of interacting proteins will be highlighted with different edge colors (blue and red) (Figure 3B), while edge width will be proportional to correlation coefficients. Furthermore, we developed a new analysis tool to show an overview of the expression correlation pattern for all pairs of interacting proteins in a given network (Figure 3E), which could help to identify the potential core module and variable components of a PPI network across tumor types.

#### Tumor type-specific prognosis biomarkers

Identifying tumor type-specific prognosis biomarkers in a PPI network is valuable to discover translational potentials. By specifying a tumor type and a threshold to dichotomize patients based on expression levels, proteins with their expression associated with significant survival difference will be highlighted in a cancer-context PPI network. Proteins associated with good prognosis (log-rank test *P*-value < 0.05, hazard ratio < 1) and poor prognosis (log-rank test *P*-value < 0.05, hazard ratio > 1) will be indicated with different node colors (Figure 3B). By clicking a node in a cancer-context PPI network, a Kaplan–Meier survival curve will be shown in the network-details panel (‘Cancer survival’ tab) to present survival differences between the patients with high-expression and low-expression of the selected protein (Figure 3F).

## DISCUSSION

PINA 3.0 is equipped with a revamped web interface, and a number of new functions to characterize the cancer contexts of human protein interactome. We hope to provide a valuable platform, to bridge the gap between cancer genomics research and PPI network analysis, by allowing users to quickly and comprehensively investigate the roles of human interacting proteins across cancer types, which has substantial functional and therapeutic significance. Although genome-wide proteomics data are currently available for a limited number of tumor samples compared to RNA-seq data, it is continuously accumulating from large International studies; thus the cancer datasets integrated in PINA will be updated quarterly to maximize its potentials. In addition, visualization and analysis tools regarding cancer functional screening and pharmacogenomic profiles will be integrated in the future to further extend its utilities in cancer research.

## Supplementary Material

gkaa1075_Supplemental_File
