# Supplementary material for: PINA 3.0: mining cancer interactome
Source: Nucleic Acids Res. 2020 Nov 24;49(D1):D1351–7. doi: 10.1093/nar/gkaa1075 (PMC7779002; doi:10.1093/nar/gkaa1075)
Supplement: gkaa1075_Supplemental_File [file gkaa1075_supplemental_file.docx]

## SUPPLEMENTARY FIGURE LEGENDS

**Supplementary Figure 1. Inferring genes with tumor type expression specificity**.

(**A**) A percent stacked barplot showing relative fractions of inferred genes in each of 33 TCGA projects with different tumor type specificity score cutoffs (mRNA level). (**B**) A histogram showing total number of genes with any tumor type expression specificity for the increasing specificity score cutoffs (mRNA level). (**C**) A histogram showing numbers of genes with expression specificity in each TCGA project with specificity score cutoff as 2 (mRNA level). (**D**) A percent stacked barplot showing relative fractions of inferred genes in each of 8 CPTAC projects with different specificity score cutoffs (relative protein abundance) (**E**) A histogram showing total number of genes with any tumor type expression specificity for the increasing specificity score cutoffs (relative protein abundance). (**F**) A histogram showing numbers of genes with expression specificity in each CTPAC project with specificity score cutoff as 0.5 (relative protein abundance).

## SUPPLEMENTARY FIGURES

**Supplementary Figure 1**

**
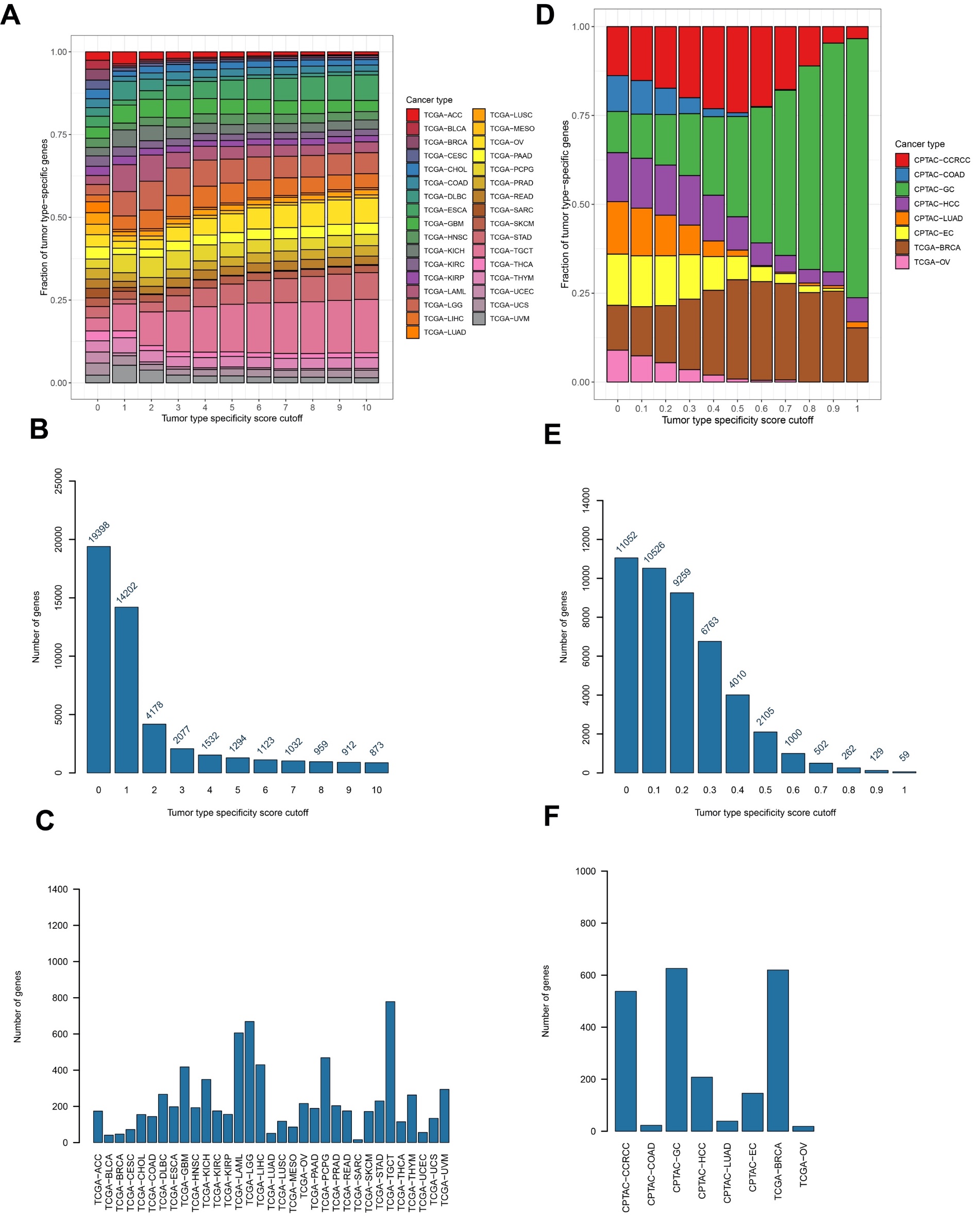
**
